# Survival and ice nucleation activity of *Pseudomonas syringae* strains exposed to simulated high-altitude atmospheric conditions

**DOI:** 10.1038/s41598-019-44283-3

**Published:** 2019-05-23

**Authors:** Gabriel Guarany de Araujo, Fabio Rodrigues, Fabio Luiz Teixeira Gonçalves, Douglas Galante

**Affiliations:** 1 Interunities Graduate Program in Biotechnology, University of São Paulo, Av. Prof. Lineu Prestes, 2415, 05508-900 São Paulo, SP Brazil; 2 Department of Fundamental Chemistry, Institute of Chemistry, University of São Paulo, Av. Prof. Lineu Prestes, 748, 05508-000 São Paulo, SP Brazil; 3 Department of Atmospheric Sciences, Institute of Astronomy, Geophysics and Atmospheric Sciences, University of São Paulo, Rua do Matão, 1226, 05508-090 São Paulo, SP Brazil; 4 Brazilian Synchrotron Light Laboratory, Brazilian Center for Research in Energy and Materials, Av. Giuseppe Máximo Scolfaro, 10000, 13083-100 Campinas, SP Brazil

**Keywords:** Atmospheric science, Air microbiology

## Abstract

*Pseudomonas syringae* produces highly efficient biological ice nuclei (IN) that were proposed to influence precipitation by freezing water in clouds. This bacterium may be capable of dispersing through the atmosphere, having been reported in rain, snow, and cloud water samples. This study assesses its survival and maintenance of IN activity under stressing conditions present at high altitudes, such as UV radiation within clouds. Strains of the pathovars *syringae* and *garcae* were compared to *Escherichia coli*. While UV-C effectively inactivated these cells, the *Pseudomonas* were much more tolerant to UV-B. The *P. syringae* strains were also more resistant to radiation from a solar simulator, composed of UV-A and UV-B, while only one of them suffered a decline in IN activity at −5 °C after long exposures. Desiccation at different relative humidity values also affected the IN, but some activity at −5 °C was always maintained. The pathovar *garcae* tended to be more resistant than the pathovar *syringae*, particularly to desiccation, though its IN were found to be generally more sensitive. Compared to *E. coli*, the *P. syringae* strains appear to be better adapted to survival under conditions present at high altitudes and in clouds.

## Introduction

The Gram-negative bacterium *Pseudomonas syringae* is a common member of epiphytic communities and an important phytopathogen in diverse crops^1^. It was the first organism found to produce biological ice nuclei (IN), being able to freeze supercooled water with exceptional efficiency at temperatures above −10 °C^2^. The IN activity of this organism originates from a large protein situated at the cell’s outer membrane, which forms multimeric clusters that structure water into an ice-like array promoting its phase change^3,4^. Homologs of this protein, called InaZ, have since been identified in other ecologically similar species also active as IN. This trait has been linked to the increased susceptibility to frost damage above −5 °C of plants harboring populations of these bacteria on their leaves, a significant concern to agriculture^5^.

The particular ability of *P. syringae* and similar organisms to form efficient IN has been long suggested to possibly influence atmospheric processes^6,7^. Glaciation, the freezing of the droplets that compose clouds, is an important mechanism leading to precipitation (including hail formation), and is largely determined by IN particles present in suspension in the air. Due to the reduced vapor pressure over ice crystals than supercooled liquid water, frozen particles can accumulate water and grow to sizes large enough to start the precipitation process inside the cloud^8,9^. This is known as the Wegener-Bergeron-Findeisen process, and its significance is evidenced by observations such as that the ice phase of clouds is the main source of rain in continental areas across the globe^10^. An additional mechanism that can amplify the influence of IN is the Hallett-Mossop process, the rapid multiplication by orders of magnitude of secondary ice crystal fragments caused by the riming and splintering of primary ice surfaces^11^. Since this occurs predominantly between −3 and −8 °C, temperatures where biological IN are the major active nuclei present in the environment^8^, this has been proposed as another potential contribution that organisms like *P. syringae* can have for the precipitation cycle^6^.

In addition to cloud glaciation, microbial cells can also exhibit activity as cloud condensation nuclei (CCN) in warm clouds^12^. These aerosol particles are essential for the condensation of water vapor into the liquid droplets that make up clouds. Interestingly, besides their IN activity, studies with bacteria of the *Pseudomonas* genus have also shown their ability to produce biosurfactants that can act as highly efficient CCN^13–15^. Further research of microbial life in the atmosphere also includes the effects of cells on cloud chemistry, particularly in relation to the metabolism of organic compounds in its aqueous phase^16^.

Multiple works have reported the presence of cultivable *P. syringae* and other ice nucleating bacteria in rain and snow samples (Morris, *et al*.^17^ and Šantl-Temkiv, *et al*.^18^, for example). Recently, Stopelli, *et al*.^19^ isolated with selective culture media IN-active *P. syringae* from snow collected at an altitude of 3,580 m at Jungfraujoch, Switzerland. Furthermore, these organisms have also been isolated directly from clouds^20,21^. Members of the *Pseudomonas* genus were the most frequently identified bacterial isolates from cloud water samples collected at an altitude of 1,465 m at the puy de Dôme summit in France between 2007 and 2010^22^. A number of *Pseudomonas* strains were also isolated from clouds and rain at the Outer Hebrides, Scotland, although those did not present IN activity^13^. These evidences point to the widespread distribution of these bacteria in the atmosphere and support their relationship with clouds and the precipitation cycle.

Besides *Pseudomonas*, a concentration of about 10^4^ total bacterial cell numbers per cubic meter of air is estimated to be found typically over land, though this figure may significantly change with the altitude, weather, season, and the underlying ecosystem^23,24^. Particles with the size of bacteria have a relatively long residence time in the air, on the order of days, during which they have the potential to cross long distances^24,25^. Effective dispersal trough this medium is, however, conditioned to cell survival as aerosols, which is a considerable challenge in this situation^26^.

The viability of aerosolized bacteria can be severely limited by atmospheric factors. Particularly, dehydration and exposure to ultraviolet (UV) light have been found to be the major barriers for successful aerial transportation of microorganisms^26^. Even inside clouds, cells are still subjected to UV, and additionally to low temperatures, freezing, and chemical stresses such as low pH and oxidizing species^16,27^. An important means of escape from this situation may be through precipitation, which can be facilitated by the IN activity of the biological particle, as mentioned above. Both field measurements and laboratory studies have shown that cells with this activity can be preferentially precipitated from clouds in this manner, more so than non-nucleating particles^19,28,29^. In this way, ice nucleation could be a valuable feature for the surviving airborne bacteria to return to the ground and again be able to multiply and propagate.

A few previous studies explored the response of *P. syringae* to simulated solar radiation in the context of atmospheric survival. Attard, *et al*.^30^ reported a reduction in viability and a somewhat small impact in IN activity of *Pseudomonas* strains after prolonged exposure to UV-A light. In contrast, Joly, *et al*.^27^ measured no loss of viability for two *P. syringae* strains exposed to a source emitting UV-B, but with a much reduced UV-A output compared to the proportion in which this wavelength range is present in the solar spectrum. IN were not evaluated in this work. Here, we improve upon these findings with a more detailed investigation of the effects of different UV ranges at a series of fluences on cell survival and ice nucleation.

Two strains of *P. syringae* were used in this study: pv. *syringae* IBSBF 281^T^ (pathovar type strain) and pv. *garcae* IBSBF 158, previously reported to be IN-active by Gonçalves and Massambani^31^. In addition to UV, the strains were also tested against desiccation at different relative humidity (RH) values (33% and <5%), a still much less understood factor for these bacteria in this context. In this manner, the aim of the experiments was to simulate particularly important^26^ individual conditions that *Pseudomonas* would be exposed to in the high atmosphere that could constrain their dispersion through the air. Nevertheless, it is worth mentioning that both tested stress factors, solar light and dehydration, can also be found at different degrees on their natural plant surface habitat. We compared the effects of distinct UV radiation sources: narrow-band UV-C and UV-B lamps, commonly used in photobiological studies, and UV-A + UV-B from a solar simulator. Previously, microorganisms have been shown to exhibit significantly different responses to these UV sources^32^, making this a novel, relevant exploration of *P. syringae* resilience. Additionally, a strain of *Escherichia coli*, another Gram-negative gamma-proteobacterium like *Pseudomonas*, but non-ice nucleation active and not a common inhabitant of plant surfaces, was used for comparison. With its status as a model organism, while not being expected to present substantial tolerance to environmental stresses present outside its usual habitat in the mammalian gut, *E. coli* is widely used for distinguishing the resistance of bacteria, often as a non-tolerant reference^33,34^. As follows, our study provides new information about microorganisms in the atmospheric environment testing varied conditions with different strains, and as can be seen next, a broad range of responses were measured.

## Results

### Ice nucleation activity of the *P. syringae* strains

**Figure 1:**
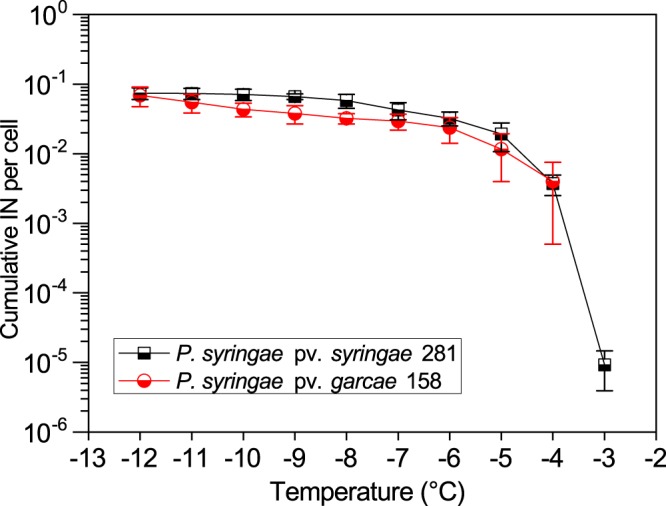
Ice nucleation spectrum of the *P. syringae* strains. Cultures of *P. syringae* pv. *syringae* 281 and *P. syringae* pv. *garcae* 158 grown in L_NP_ medium at 15 °C were tested with the droplet freezing assay to quantify their ice nuclei (IN) concentration at decreasing temperatures. The data points at warmer temperatures represent the initial temperature at which frozen droplets could be detected for each strain. Error bars denote standard deviations of the means (n = 5).

Ice nucleation profiles across a range of temperatures were determined by the droplet freezing assay^35^ for the *P. syringae* strains (Fig. 1). Cells were grown in nutrient-limited L_NP_ medium^36^ and at cultivation conditions chosen to allow for maximum expression of the IN phenotype. Different to what was previously reported by Gonçalves and Massambani^31^, the pv. *syringae* strain (281) had a stronger measured IN activity than the pv. *garcae* strain (158). No IN active at −3 °C could be reliably detected for 158, while 9 ± 5 × 10^−6^ nuclei per cell could be measured at this temperature for 281. Despite that, the overall IN spectrum of both strains was mostly similar for the remaining temperatures tested. For the other experiments, only the nuclei concentration at −5 °C was chosen to be studied. This temperature was used for being at the lower range for maximum environmental significance of the IN activity, be it for causing frost damage on leaves^5^ or affecting cloud glaciation^6,11^.

### *P. syringae* survival and ice nucleation activity following exposure to different UV ranges

**Figure 2:**
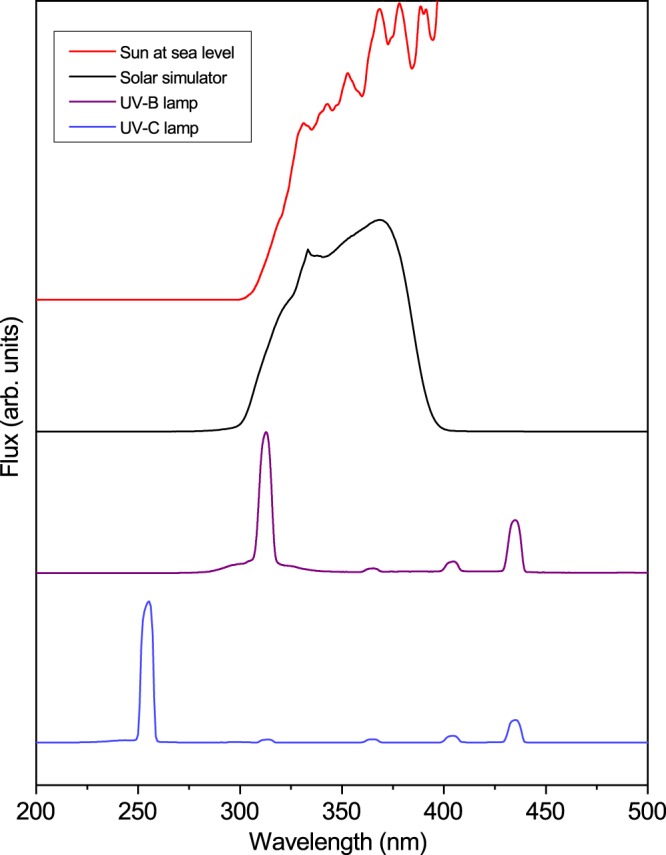
Comparison of the spectra of the lamps used for the experiments. From top to bottom (in arbitrary flux unities): the Sun over Earth’s surface (ASTM G173-03, smoothed), the Oriel Sol-UV-2 solar simulator, and the UV-B and UV-C lamps, measured with an Ocean Optics QE65000 spectrometer. It is apparent that the commonly used UV-B and UV-C narrow-band lamps largely differ from the UV in the real environment.

**Figure 3:**
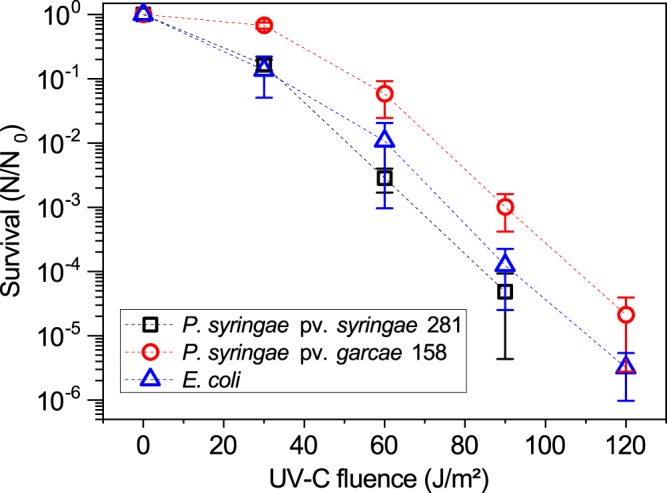
Survival curves to UV-C (254 nm) radiation. *P. syringae* strains were exposed as 5 × 10^6^ cells/ml suspensions, and *E. coli* as 5 × 10^7^ cells/ml. *P. syringae* pv. *syringae* 281 and *P. syringae* pv. *garcae* 158 are shown to be as sensitive as *E. coli* to this UV range often used in laboratory experiments but absent on Earth’s surface. Error bars denote standard deviations of the means (n = 3).

The UV resistance of the microorganisms was first tested with common laboratory narrow-band (monochromatic) UV-C and UV-B sources. Spectra of all the lamps used for the experiments can be observed in Fig. 2, and fluence values and intensities were measured by a Vilber Loumart radiometer (RMX-3W, Marne-la-Vallée, France). The survival curve to UV-C radiation (254 nm) of *P. syringae* pv. *syringae* 281 remained within 1 standard deviation of *E. coli*, while *P. syringae* pv. *garcae* 158 presented a generally slightly higher mean survival but still overlapped with *E. coli* at the final 120 J/m^2^ point (Fig. 3). Under our tested conditions, at a fluence of 60 J/m^2^, the CFU counts of 281 were reduced to 0.3 ± 0.1% of the initial population, which was of about 5 × 10^6^ cells/ml for both strains for all experiments. This can be compared to 158, for which this UV-C fluence caused just over a 1 log decrease in viability (6 ± 3% survival). In any case, both *Pseudomonas* can be considered sensitive to this radiation range. It is important to note, though, that solar UV-C is mostly absent below the highest reaches of the atmosphere.

**Figure 4:**
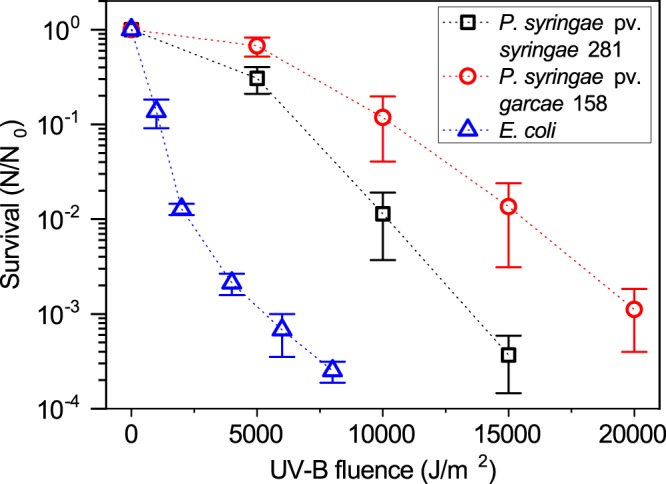
Survival curves to UV-B (312 nm) radiation. *P. syringae* strains were exposed as 5 × 10^6^ cells/ml suspensions, and *E. coli* as 5 × 10^7^ cells/ml. *P. syringae* pv. *syringae* 281 and *P. syringae* pv. *garcae* 158 are shown to tolerate much larger UV-B fluences than *E. coli*, while 158 presented the highest resistance. Error bars denote standard deviations of the means (n = 4).

For the UV-B (312 nm) assays, the observed survival of the *P. syringae* strains was considerably greater than *E. coli* (Fig. 4). At a fluence of 5,000 J/m^2^, *E. coli* was inactivated by nearly 3 orders of magnitude, while both 281 and 158 still retained 31 ± 10% and 67 ± 15% of viability, respectively. Treatment with higher fluences (up to 20,000 J/m^2^) evidenced a larger UV tolerance of 158 compared to 281, which was inactivated by over 4 logs at this final point (not being reliably recovered for proper quantification). Despite being present in the environment, the UV-B of the narrow-band lamps poorly represents the real solar spectrum. For a more faithful recreation of UV in Earth’s atmosphere, an Oriel solar simulator (Sol-UV-2, Bozeman, USA) was used (Fig. 2).

**Figure 5:**
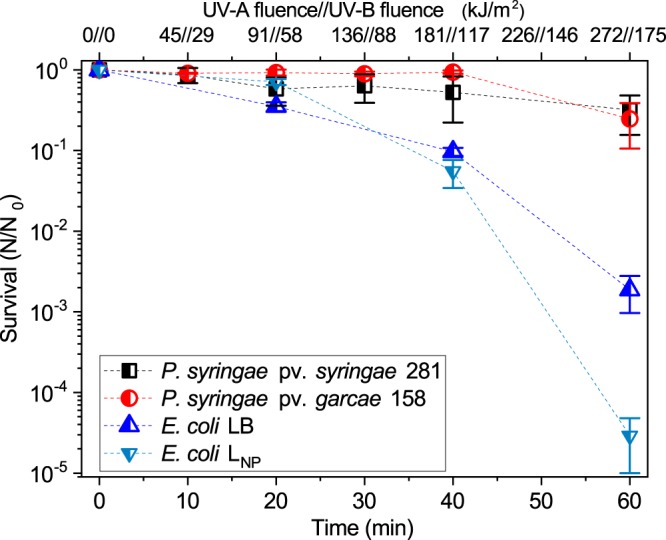
Survival curves to “environmental” UV radiation (UV-A + UV-B). For this more accurate representation of the solar UV found in the troposphere, *P. syringae* pv. *syringae* 281 and *P. syringae* pv. *garcae* 158 suffered a decrease in viability of less than 90% after 60 minutes of exposure, from an initial population of ~5 × 10^6^ cells/ml. Meanwhile, LB-grown *E. coli* presented a 1,000-fold reduction in survival from its initial numbers of ~5 × 10^7^ cells/ml. When grown in L_NP_ , the minimal medium used for the *P. syringae* strains, *E. coli* was even more sensitive, with a drop in viability after the full exposures of almost 5 logs (from about 10^7^ cells/ml in this case). Error bars denote standard deviations of the means (n = 3).

For the “environmental” UV experiments with the solar simulator, cell suspensions were exposed placed on top of a frozen foam block to avoid evaporation and excessive heating. The intensities used were 75.5 W/m² for the UV-A and 48.7 W/m² for the UV-B at the ranges measured by the radiometer. This UV-B value, which can be taken as the more biologically relevant region of the spectrum, is equivalent to about 5.2 times the intensity at an altitude of 850 m in São Paulo, Brazil (9.3 W/m²) and 3.1 times the value at an altitude of 5091 m at the Atacama Desert, Chile (15.6 W/m²), as reported in a previous paper^32^. The *Pseudomonas* strains were found to be significantly more tolerant than *E. coli* to this “environmental” UV range (Fig. 5). A 60 minute exposure, equivalent to up to several hours in the real environment, did not reduce by more than 1 log the viability of 281 (32 ± 16% survival) or 158 (25 ± 14%), while *E. coli* survived at only 0.2 ± 0.1%. Interestingly, the response of both *P. syringae* strains, for which survival curves were nearly overlapping, was much more similar to the UV-A + UV-B used for these experiments than for the monochromatic UV-B (Fig. 4), again evidencing the biological relevance of differing spectra.

To test if the *Pseudomonas* culture conditions somehow favored the greater survival of these cells in relation to LB-grown *E. coli*, further experiments were done. *E. coli* cultivated in nutrient-poor L_NP_ medium, the same used for the *P. syringae* strains, was tested at the solar simulator. Remarkably, its survival after one hour of irradiation, 0.003 ± 0.002%, was even inferior to cells grown in rich medium at the same fluences (0.2 ± 0.1%), a difference of almost 2 logs (Fig. 5). In this manner, a poorer growth medium was not observed to contribute to UV tolerance, and, in fact, actually resulted in more sensitive cells.

**Figure 6:**
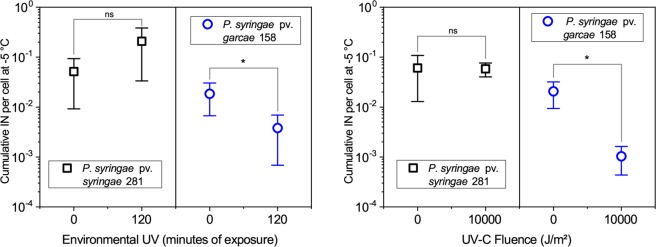
IN activity following prolonged exposures to “environmental” UV radiation (UV-A + UV-B) (right panel) and to UV-C (254 nm) radiation (left panel). For the “environmental” UV, when compared to non-irradiated controls (“0 min”), the concentration of cumulative ice nuclei (IN) per cell at −5 °C of *P. syringae* pv. *syringae* 281 exposed for two hours suffered no significant change, while *P. syringae* pv. *garcae* 158 samples exhibited an up to 10-fold reduction. The IN of *P. syringae* pv. *syringae* 281 exposed to 10,000 J/m^2^ of UV-C remained stable when compared to non-irradiated controls (“0 J/m^2^”). *P. syringae* pv. *garcae* 158, instead, was reduced by about 20-fold. Error bars denote standard deviations of the means (n = 7 for 281 and n = 5 for 158 for the “environmental” UV experiments, n = 6 for 281 and n = 7 for 158 for the UV-C experiments). Based on the Mann-Whitney U test, significantly different values are marked as *(p < 0.05) and non-significant differences as “ns”.

The cumulative IN activity of the cells at −5 °C was measured after irradiation for a much longer period (120 minutes) of simulated “environmental” UV (Fig. 6). The strain 281 exhibited no significative difference from its initial cumulative IN concentration when assayed after the exposure. Meanwhile, 158 presented an up to 10-fold decrease from the typical 10^−2^–10^−1^ nuclei per cell of this strain at this same temperature (seen in the “0 min” control and also in Fig. 1). Despite that, the *Pseudomonas* ice nuclei endured UV even better than the cells themselves. Experiments with very large UV-C fluences (10,000 J/m^2^), far beyond the point where no surviving cell could be expected (see Fig. 3), also yielded no difference in 281’s IN at −5 °C, and a reduction by only about 20 times for 158 (Fig. 6), further confirming this observation.

### Survival and ice nucleation activity of desiccated cells

**Figure 7:**
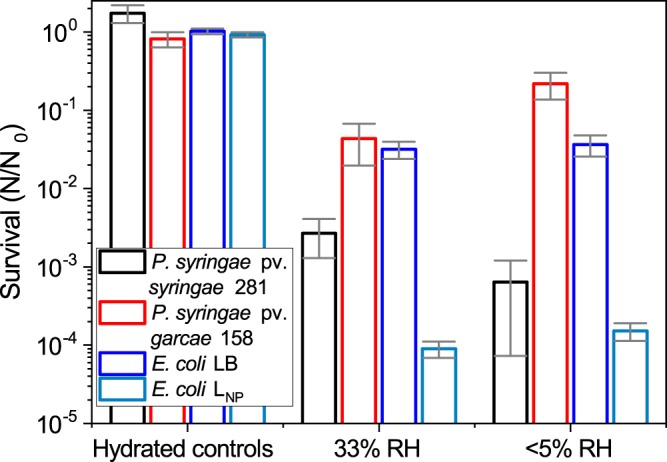
Desiccation tolerance at different relative humidity (RH) values. Survival of hydrated controls and samples desiccated at 33% and <5% RH for 6 days at 20 °C of *P. syringae* pv. *syringae* 281, *P. syringae* pv. *garcae* 158, and *E. coli* grown either on LB or L_NP_ are presented. The 281 strain is shown to be sensitive to dehydration, while 158 is considerably more tolerant to this treatment. *E. coli* tolerated relatively well the desiccation, but when grown in L_NP_ minimal medium these cells were inactivated by additional 3 logs. Error bars denote standard deviations of the means (n = 4).

For the desiccation assays, cells were dried inside microcentrifuge tubes for 6 days at 20 °C, or kept hydrated in NaCl 0.9% solution as controls. During this period, the tubes were stored inside containers with controlled relative humidity (RH), using MgCl_2_ for 33% RH and silica gel for <5% RH. Those results are presented in Fig. 7. The largest tolerance was exhibited by the 158 strain at <5% RH, in which 22 ± 8% of its initial population of 5 × 10^6^ cells survived. At the same treatment, the viability of 281 was reduced by 10^3^–10^4^ times (also from 5 × 10^6^ cells). At 33% RH, the surviving percentage of 158 was 4 ± 2%, about 10 times more than 281. For both tested RH (33% and <5%), *E. coli* mean survival was around 3 to 4% (from 5 × 10^7^ cells). As can be observed, 158 presented some degree of tolerance to desiccation, while 281 was much more sensitive. Comparing between organisms, no clear tendency is apparent in relation to RH and cell survival. Hydrated control samples remained mostly at the same initial CFU concentration, except for 281 for which the number of cells increased slightly. Again, for comparison, *E. coli* grown in L_NP_ minimal medium was tested. It presented a far reduced survival, with a decrease of over 3 orders of magnitude in relation LB-grown cells (from about 10^7^ cells, Fig. 7).

**Figure 8:**
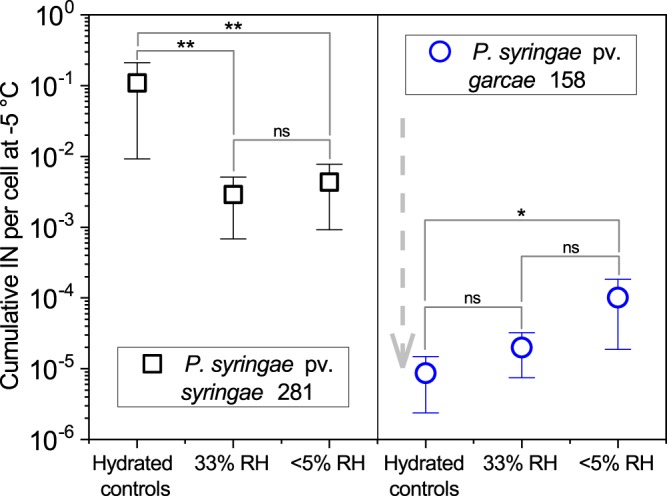
IN activity following desiccation. The concentration of cumulative ice nuclei per cell at −5 °C of *P. syringae* pv. *syringae* 281 presented a significant (p < 0.01) decrease after desiccation at 33% and < 5% RH for 6 days at 20 °C compared to its hydrated controls. Surprisingly, *P. syringae* pv. *garcae* 158 suffered a large reduction in IN in relation to its typical values (repeatedly measured as 10^−2^–10^−1^ per cell at −5 °C, Fig. 1) even while hydrated, as indicated by the downwards grey arrow. Error bars denote standard deviations of the means (n = 6). Based on Bonferroni-corrected Mann-Whitney U tests, significantly different values are marked as *(p < 0.05) or as **(p < 0.01), and non-significant differences as “ns”.

The IN activities of the desiccated *P. syringae* strains were strikingly different (Fig. 8). After being resuspended from the RH 33% and RH <5% treatments, the 281 strain presented a reduced mean cumulative nuclei concentration of about 3–4 × 10^−3^ per cell at −5 °C. Probably, this can be attributed to the disruption of the cells’ outer membranes where the ice nucleation protein clusters lie. Its hydrated controls remained at typical values for these cultures, at 1.1 ± 1.0 × 10^−1^. However, the IN concentrations for the 158 strain, which are normally similar to those of 281 (see Fig. 1), were lower by 10^3^–10^4^ times, even for hydrated cells that kept most of their viability. Its mean measured IN activities (concentration per cell at −5 °C) ranged from 1 × 10^−4^ to 9 × 10^−6^, after the 6-day period at 20 °C. Since *Pseudomonas* IN are known to be heat-sensitive, the temperature was suspected to play a role in this decline, so new experiments were performed storing the 158 strain samples at 4 °C. Survival was very similar at these conditions, and a substantially increased maintenance of IN at the hydrated control and RH 33% samples was observed, while the RH <5% treatment remained mostly identical (Supplementary Fig. S1). Yet, these values were still lower than the typical IN concentration for this strain (Fig. 1).

## Discussion

Both *P. syringae* strains were found to be very sensitive to UV-C, exhibiting survival comparable to the non-UV tolerant bacterium *E. coli* (Fig. 3). This range of Solar radiation (<280 nm) does not reach Earth’s lower atmosphere (the troposphere), being completely absorbed at the stratosphere. Despite that, the 254 nm wavelength produced by low-pressure mercury lamps is widely used to assess the effects of UV on bacteria in laboratory studies. It efficiently damages DNA and inactivates microorganisms, and, as such, is commonly referred to as “germicidal UV”^37^. Nevertheless, some bacteria, such as *Deinococcus radiodurans*, show extreme tolerance to UV-C^38^. For comparison, a UV-C fluence of about 1,000 J/m^2^ is required to inactivate 90% of its population^32^, while the highest fluence tested in this work was just 120 J/m^2^ (Fig. 3). The biological effects of the different UV radiation ranges and known UV tolerance systems are presented in the Supplementary Discussion.

Covering the wavelengths between 280 and 320 nm, UV-B is the most energetic, and potentially damaging, range of solar radiation that reaches the ground. At higher elevations, the UV flux is increased in relation to lower altitudes, more so at the UV-B region^39^. In agreement to that, Wang, *et al*.^40^ measured a more severe DNA damage in plants at an altitude of 1,700 m than at 300 m. In contrast to the UV-C assays, the *P. syringae* strains were significantly more resistant to this UV range (at 312 nm) than *E. coli* (Fig. 4). Even then, it must be recognized that these organisms are comparatively much less tolerant than *D. radiodurans*, for which over 120,000 J/m^2^ of UV-B is required to inactivate about 90% of its population at identical experimental conditions^32^. Still, these results point to some degree of resistance of *P. syringae* to UV-B, and it is important to notice that these cells are naturally exposed to a much different spectrum which effects considerably different responses^32^.

The strain 158 (*P. syringae* pv. *garcae*) was more tolerant than 281 (*P. syringae* pv. *syringae*) to the UV-B at the higher fluences tested. This evidences substantial differences between the strains which can also be observed for the other experiments discussed further below, despite both being classified as the same species. In fact, *P. syringae* pv. *garcae* can be distinguished as belonging to a separate, discrete *genomospecies* than *P. syringae* pv. *syringae* on the basis of DNA differences, though a lack of discerning phenotypic characteristics has prevented its reclassification with other strains within the “*P. coronafaciens*” taxon^41^. This fact may explain the observations made in this study, where the distinct measured phenotypes probably reflect the underlying dissimilar genotypes.

For a more accurate, relevant representation of the environmental UV than the narrow-band lamps, a solar simulator emitting UV-A + UV-B was used (Fig. 2). Under these conditions, the *P. syringae* strains were shown to be much more resistant than *E. coli* (Fig. 5). This can be partially seen as a consequence of the observed higher tolerance of *Pseudomonas* to the UV-B (Fig. 4), though the presence of UV-A (320–400 nm) can significantly affect some organisms. For example, *D. radiodurans* is surprisingly sensitive to this higher wavelength fraction of the environmental UV^32,38^. This confirms that *P. syringae* can be considered truly tolerant to solar radiation. Additionally, it is important to note that, due to differences between the UV-B lamps’ line emission at 312 nm and the solar simulator broad spectrum (~290–400 nm, Fig. 2), the biological response to both sources is not exactly equivalent. Thus, the survival curves presented for UV-B and “environmental” UV cannot be directly compared on the basis of the measured fluences, considering the Vilber Loumart radiometer’s probes read a spectral range, not a single wavelength.

Joly, *et al*.^27^ exposed two *P. syringae* isolated from cloud water for 10 hours to total final fluences of 85.7 kJ/m^2^ of UV-A and 27 kJ/m^2^ of UV-B at 5 °C. After this period, the isolates suffered virtually no viability loss. In the results presented in Fig. 5, it is revealed that *Pseudomonas* strains actually tolerate acute expositions to considerably larger fluences than the ones used for this previous study. Still, these authors used a lamp with a much different spectrum from the solar simulator, with proportionally less UV-A and a large amount of visible light. Attard, *et al*.^30^ used a 1,000 Watt xenon lamp filtered by a glass lid with a total UV-A intensity of 33 W/m^2^. Interestingly, exposure for 42 hours in distilled water at 17 °C, only reduced the viability of three different *P. syringae* isolates by about 90% in relation to non-irradiated controls. UV-B measurements (if significant) were not provided, but the final calculated UV-A exposure was of almost 5,000 kJ/m^2^. Possibly, this less acute exposition could have improved the tolerance of the strains, considering the lower intensity and absence of the more damaging UV-B wavelengths.

As mentioned, the UV-B intensity used for the “environmental” UV experiments is much higher than the values found in some natural environments, even at high altitudes, as reported previously^32^. Those measurements were performed by the same probes used for the solar simulator with a Vilber Loumart radiometer, under clear sky conditions, at noon, during summer, and at similar latitudes. Taking the UV-B as the most biologically relevant radiation range for comparison, *P. syringae* strains can be expected to tolerate hours of direct sunlight exposure, even without attenuating factors such as association to cell clusters, mineral particles or organic fragments. Certainly, those factors can become increasingly important for survival at higher altitudes and for longer periods.

The IN activity of 281 was not reduced for cells irradiated for two hours under the solar simulator. The 158 strain, instead, suffered an up to 10-fold decrease at −5 °C, showing that UV can have at least some effect on this trait (Fig. 6). The *P. syringae* isolates irradiated with UV-A by Attard, *et al*.^30^ (mentioned above) presented either a non-significant difference in IN activity at −5 °C or suffered a small reduction. These authors discuss how dead bacteria could maintain their IN activity as long as cell integrity is not disrupted, preserving the large ice nucleation protein aggregates on the cells’ outer membranes. In fact, this is what was observed for cells completely inactivated by UV-C exposure, with again only a relatively small effect measured for 158 (Fig. 6).

Summarizing the UV results, our work reveals that the biological response to commonly used laboratory UV sources is a poor representation of what is found with the solar spectrum, and that while UV can be a serious limitation to the dispersal of *P. syringae* through the atmosphere, these bacteria are adapted to endure periods of complete exposure to sunlight. A relatively large subset of their population remained capable of influencing cloud nucleation, which, in turn, could favor their deposition back to the ground^19,28,29^. Perhaps, a greater challenge to these cells is desiccation, both in relation to survival and IN maintenance (see Figs 7 and 8).

Dehydration of cells causes membrane damage, DNA strand breakage, and an increased formation of reactive oxygen species from the cellular metabolism leading to protein oxidation^42,43^. Similarly to the UV (see Supplementary Discussion), DNA repair and antioxidant systems should be valuable features for bacterial survival under this condition. In the environment, tolerance to desiccation could possibly be achieved by activation of the aerosolized bacteria as cloud condensation nuclei (CCN), enabling hydration of the cells from water vapor harnessed from the air. Since *Pseudomonas* were shown to be potential efficient CCN due to biosurfactant production^13–15^, this could be another survival mechanism at the disposition of these cells.

Cell clustering could be an important factor contributing to *P. syringae* tolerance to desiccation when airborne. The experiments were conducted in a manner so as to minimize these artifacts, exposing cells as thin layers to rapidly equilibrate with the RH controlling agents, but masses of natural bacterial populations are probably better adapted to endure this stress. Monier and Lindow^44^ have previously shown that cells grown as aggregates over leaf surfaces present an increased desiccation tolerance with larger cells clusters, but artificially generated aggregates did not exhibit such effect. An explanation for that is the lack of locally-produced extracellular polymeric substances (EPS), which are formed by cells grown together as groups, but would not be extensively produced under the laboratory culture conditions used. In addition to this point, the osmotic shock of rehydration after desiccation under the tested laboratory conditions could be a significant stress by itself. As reported by Kosanke *et al*.^45^, a slow rehydration protocol can increase the survival of dried bacteria. Despite this effect being relatively minor when compared to the large range of survivals measured in this work, it could have also played a role in the results obtained.

Despite its low tolerance to desiccation, the IN activity of 281 was relatively well preserved after desiccation (Fig. 8). The measured decrease by roughly 30 times in IN concentration can possibly be attributed to cell membrane disruption during dehydration affecting the IN protein clusters. Its hydrated controls maintained the full typical IN concentration of this strain (about 10^−1^ per cell). In contrast, the IN activity of the more desiccation tolerant strain 158 was unexpectedly reduced even in the hydrated controls (Fig. 8) when compared to its typical IN concentration (Fig. 1). The large aggregates of InaZ proteins that form at the cells outer membranes, which are essential for forming efficient IN active at relatively high temperatures such as −5 °C, are known to be particularly heat sensitive^36^. Even though the temperature of 20 °C at which the hydrated and desiccated cells were kept at during these experiments were not expected to be detrimental to biological IN^36^, further tests with 158 were performed at 4 °C (Supplementary Fig. S1**)**. This could not recover the typical IN concentration for this strain (Fig. 1), signifying some other mechanism contributes to the instability of the nuclei in this case where the cells are not actively growing and their metabolism, including protein synthesis, is probably reduced. Nevertheless, it can be summarized that one of our main findings is that desiccation can be a much more severe limitation to cells and IN than UV.

Aerial emission of *P. syringae* from plants was observed to be greater at dryer conditions on sunny days^46,47^. Despite the abundance of cells being lifted into the air, these desiccating circumstances may impart a significant limitation on living cell dispersion and IN activity right from the start. Alternatively, different studies^48–50^ found large increases of airborne IN evidenced to be to be of microbial origin during and following rainfall. The actual mechanism of aerosolization from raindrop impact has only recently began to be understood^51^, but these wet conditions may greatly enhance the efficiency of bacterial dissemination through the air within droplets (or at least at high humidity). This may be important for research on feedback effects between rain and IN. Precipitation events may increase further rain by seeding the atmosphere with suspended biological particles^52^, and the likely enhanced survival and IN preservation when compared to dry, desiccating conditions may be a key step enabling this cycle. Further studies will be necessary to make clear how the biological response of bacterial cells to environmental stresses can have further consequences to the ecosystem they inhabit.

## Material and Methods

### Strains, media, growth conditions, and survival quantification

The tested *Pseudomonas syringae* belonged to the strains IBSBF 281^T^ (=NCPPB 281, ATCC 19310; pv. *syringae* type strain; isolated from *Syringa vulgaris*) and IBSBF 158 (pv. *garcae*; isolated from *Coffea arabica*, where it causes the brown spot disease on leaves). For maximum expression of the IN phenotype, cells were grown at 15 °C in L_NP_ medium (3-(N-morpholino)propanesulfonic acid (MOPS), 10.46 g L^−1^; KCl, 1.86 g L^−1^; NH_4_Cl, 0.11 g L^−1^; Na_2_SO_4_, 1.42 g L^−1^; NaCl, 0.58 g L^−1^; MgCl_2_·6H_2_O, 0.20 g L^−1^; KH_2_PO_4_, 0.014 g L^−1^; CaCl_2_, 0.011 g L^−1^; FeCl_3_·6H_2_O, 0.0027 g L^−1^; sorbitol, 4 g L^−1^; pH 7.2^36^) for 3 days to an OD of ~0.5. Colony-forming units (CFU) were enumerated on Difco Nutrient Agar added with 2.5% glycerol (NAG) plates incubated in the dark at 20 °C. *E. coli* BL21 was cultivated at 37 °C in Difco LB Broth. Cultures were grown overnight to an OD of ~5.0 in LB, and enumerated on LB agar plates incubated in the dark at 37 °C. For some experiments, *E. coli* was grown in L_NP_ medium overnight at 37 °C to an OD of ~0.5. All cell suspensions were diluted in saline solution (NaCl 0.9% w/v) prepared with ultrapure Milli-Q water (Millipore, Molsheim, France). Survival is expressed as the fraction N/N_0_, where “N” is the dilution-corrected number of UFC recovered after each treatment and “N_0_” is the number of initial UFC from before the experiments. Survival fraction values are presented as means of the replicates, with error bars denoting standard deviations.

### Quantification of ice nucleation activity

Ice nucleation activity for each sample was quantified by the droplet freezing assay, using diluted cell suspensions placed as arrays of 32 drops of 10 μl on top of a paraffin-coated aluminum tray. This coating was previously applied as a 2% solution of paraffin in xylene, with the solvent removed by heat over a hot plate. The tray was covered with a transparent acrylic lid sealed on the borders by a ring made of EVA foam sheet and held in place by binder clips. This set was then positioned almost totally immersed in a low temperature circulating bath (Neslab LT-50, Newington, USA) filled with 96% ethanol. Temperature was monitored with a submerged mercury thermometer. From −2 °C, the bath temperature was reduced in 1 °C stages, which were held for at least 5 minutes. At each stage, the number of frozen drops was scored. IN concentration was calculated with the following equation adapted from Vali^35^, as commonly used for microbiological studies (e.g., Joly, *et al*.^21^): c(T) = [ln (N) − ln (N − N(T))]/A, where “c(T)” is the number of cumulative active IN per cell at temperature “T”, “N” is the number of drops tested, “N(T)” is the number of frozen drops at temperature “T”, and “A” is the number of cells per drop (determined by CFU counting from initial population). All experiments were replicated at least 4 times. Measured IN activity values are presented as means of the replicates, with error bars representing standard deviations. To determine significant differences, Mann-Whitney U tests and Bonferroni corrections were performed with PAST (v3.21) software^53^ (http://folk.uio.no/ohammer/past/).

### UV irradiation experiments

UV-C irradiation was done with a Philips TUV-20W low-pressure mercury lamp with main emission line at 254 nm. UV-B was provided by a set of mercury lamps (two LightTech Narrow Band UV-B 20 W and one Philips TL 20 W/01 RS) with main emission line at 312 nm. A solar simulator (Oriel Sol-UV-2, Bozeman, USA) with a 1,000 Watt xenon arc lamp was used for the “environmental UV” irradiation experiments. This source’s output covers partially the UV-B and UV-A ranges with a spectrum similar to the one found at directly Sun-exposed environments on Earth (with negligible UV-C), except most of the visible light is removed by an optical filter (Fig. 2). Irradiation intensities and fluences were measured by a radiometer (Vilber Loumart RMX-3W, Marne-la-Vallée, France) with photocells specific to wavelength ranges centered in 254 nm at the UV-C (CX-254), 312 nm at the UV-B (CX-312), and 365 nm at the UV-A (CX-365).

For the UV-C and UV-B assays, cultures were diluted 100-fold in saline solution to a final volume of 8 ml in autoclaved 7 cm diameter glass petri dishes (without the lids). The samples were irradiated under orbital shaking while the fluences were monitored in real time by photocells placed next to the dishes. Aliquots taken at fluence intervals were diluted and plated for survival quantification by enumeration of CFU. For the “environmental” UV experiments, the samples were irradiated on top of a frozen foam block, also under shaking, so as to control their temperature to near 0 °C. This was done during these more prolonged exposures to avoid evaporation and to prevent excessive heating – which can itself affect the cells’ IN by leading to the disaggregation of the ice nucleation protein clusters on the cells’ outer membranes^36^. The intensities were measured beforehand as 75.5 W/m² for the UV-A and 48.7 W/m² for the UV-B, at the ranges read by the radiometer (centered on 365 and 312 nm). Aliquots from the exposed samples were then taken after determined time intervals, corresponding to UV-A and UV-B fluences which could be calculated afterwards from the intensity values.

Separate experiments were performed to test the effects of UV over the cells’ IN. Longer exposures were used in these assays since preliminary trials showed no effects of smaller fluences on IN activity. With the solar simulator, samples were exposed for 120 minutes at the same UV intensities as before, equivalent to a total 545 kJ/m² of UV-A and 348 kJ/m² of UV-B (at the ranges read by the radiometer), twice as much as the largest fluence of the survival tests. In this case, the diluted cultures were exposed as 2 ml volumes in 3 cm diameter dishes, allowing more samples to be placed below the source’s focus at the same time. The control samples, “0 min”, were aliquoted from the dishes before the beginning of the experiments and stored until the end of the irradiation when their IN activity was quantified parallel to the “120 min” samples.

### Desiccation experiments

Desiccation assays were performed with 10 μl volumes taken directly from the cultures and deposited in the internal wall of horizontally positioned autoclaved 1.5 ml microcentrifuge tubes. The tubes were then placed inside sealed recipients containing either silica gel beads or water-saturated MgCl_2_. These treatments were used to provide controlled relative humidity (RH) values below 5% and of about 33%, respectively^54^. A <5% RH is typical for very high altitudes, considering water vapor sources at the ground surface. Hydrated controls were prepared by adding 10 μl from the cultures to a total 1 ml in tubes with saline solution (10^−2^ dilution). All samples were stored for 6 days inside an incubator at 20 °C. During this, the temperatures were monitored with mercury thermometers and were found to remain stable. At the end of this period, the dried samples were resuspended with 1 ml saline solution (10^−2^ dilution in relation to the cultures). Along with the hydrated control cell suspensions, these tubes were diluted for survival determination by plating and for IN quantification. Survival was calculated relative to initial controls aliquoted from the cultures, plated before the beginning of each experiment.

## Supplementary information

Supplementary Information
