# Supplementary material for: Survival and ice nucleation activity of Pseudomonas syringae strains exposed to simulated high-altitude atmospheric conditions
Source: Sci Rep. 2019 May 23;9:7768. doi: 10.1038/s41598-019-44283-3 (PMC6533367; doi:10.1038/s41598-019-44283-3)
Supplement: Supplementary file 1 — Supplementary Information [file 41598_2019_44283_MOESM1_ESM.pdf]

SUPPLEMENTARY INFORMATION

Survival and ice nucleation activity of *Pseudomonas syringae* strains exposed to simulated high-altitude atmospheric conditions

Gabriel Guarany de Araujo[a], Fabio Rodrigues[b], Fabio Luiz Teixeira Gonçalves[c], Douglas Galante[d,*]

[a] Interunities Graduate Program in Biotechnology, University of São Paulo, Av. Prof. Lineu Prestes, 2415, 05508-900, São Paulo, SP, Brazil

[b] Department of Fundamental Chemistry, Institute of Chemistry, University of São Paulo, Av. Prof. Lineu Prestes, 748, 05508-000, São Paulo, SP, Brazil

[c] Department of Atmospheric Sciences, Institute of Astronomy, Geophysics and Atmospheric Sciences, University of São Paulo, Rua do Matão, 1226, 05508-090, São Paulo, SP, Brazil

[d] Brazilian Synchrotron Light Laboratory, Brazilian Center for Research in Energy and Materials, Av. Giuseppe Máximo Scolfaro, 10000, 13083-100, Campinas, SP, Brazil

* Corresponding author

SUPPLEMENTARY FIGURES

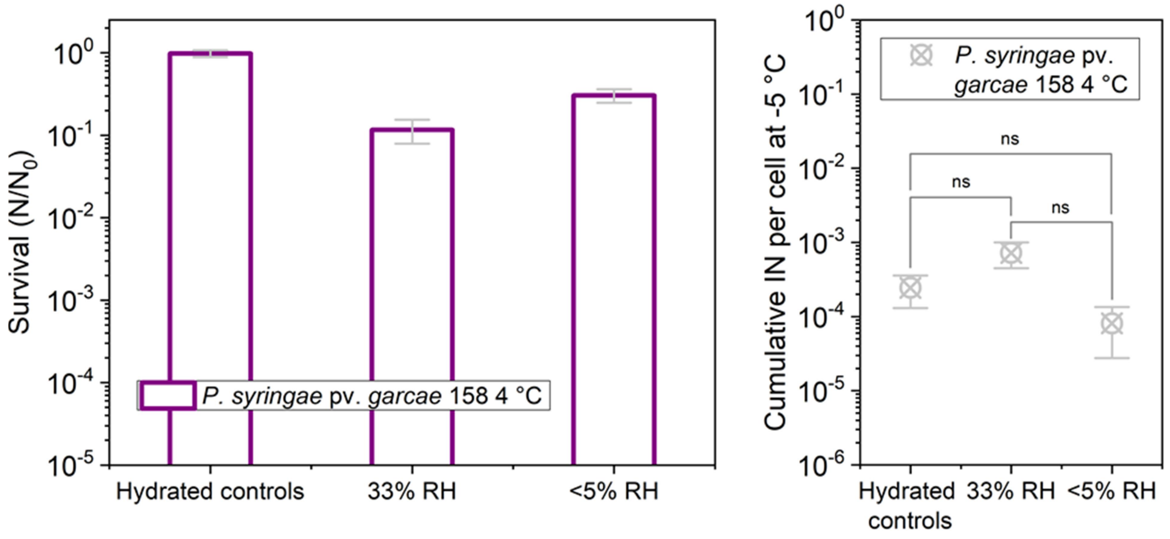

Supplementary Figure S1. Desiccation of *P. syringae* pv. *garcae* 158 at 4 °C. Survival of samples desiccated at 33% and <5% RH for 6 days inside a refrigerator (left panel) was nearly identical to what was observed at 20 °C (**Figure 7**). The concentration of cumulative ice nuclei per cell at -5 °C (right panel) was still much reduced in relation to the typical values for this strain (**Figure 1**), similar to what was observed in the treatments at 20 °C (**Figure 8**). Error bars denote standard deviations of the means (n=4). Based on Bonferroni-corrected Mann-Whitney U tests, all differences in the IN experiments were found to be non-significant and thus were marked as “ns”.

SUPPLEMENTARY DISCUSSION

Biological effects of UV and UV tolerance mechanisms.

UV-C causes the photochemical formation of dimers between adjacent pyrimidine bases on the cells' DNA. The main photoproducts generated are cyclobutane pyrimidine dimers (CPDs) and (6-4) pyrimidine-pyrimidone photoproducts (6-4 PPs), which block transcription and DNA replication with potential lethal effects for the cell [1]. A widespread UV resistance mechanism is photorepair, where a specific enzyme, called photolyase, binds pyrimidine dimers in the cell's DNA and uses luminous energy (UV-A or visible light) to independently repair the damage. Otherwise, cells may rely on the nucleotide excision repair (NER) pathway, a multi-step (and more energetically demanding) enzymatic process that removes a patch of the lesioned strand that is subsequently resynthesized [1-3]. The organisms tested in this work all probably possess these very common repair pathways, including other relevant systems such as base excision repair (BER) and homologous recombination, which have already been characterized in *E. coli* and can be identified in *P. syringae* genomes [4].

In addition to DNA repair, it was found that protection of cellular proteins from reactive oxygen species (ROS) generated upon exposure to UV-C is an important factor for the survival of *D. radiodurans* to this condition [2,5]. This was compared to *E. coli*, which is UV-sensitive and whose proteome is severely oxidized by UV-C. Its damaged repair machinery is prevented from correcting DNA injuries and lead, ultimately, to cell death [5]. In this manner, avoidance of ROS formation and effective quenching of these species represent another important UV tolerance mechanism. Still, different UV wavelengths induce distinct biological effects [6], which may require specific adaptations to allow the survival of the irradiated organism.

UV-B is known to directly create DNA photoproducts, like UV-C, but is also capable of causing significant oxidative damage from ROS [6]. Some *P. syringae* strains possess the error-prone DNA polymerase V encoded by the *rulAB* operon, which is responsible for translesion synthesis over damaged DNA template strands. The expression of this polymerase is induced by UV-B and confers increased resistance towards irradiation at the cost of increased mutability [7]. This operon is most commonly found in plasmids and its occurrence is variable within the species, even in strains of the same pathovar [4,8,9]. Its presence in the *Pseudomonas* strains tested in this work would thus have to be individually verified if this tolerance factor was to be attributed to them.

The deleterious biological effects of the UV-A range are mostly linked to ROS production, damaging, albeit indirectly, the cells' DNA, protein, and lipids [6]. Even so, though much less efficiently than UV-B, UV-A is also able to form CPDs, and it can additionally cause the photoisomerization of 6-4 PPs into its Dewar valence isomers, another type of environmentally-relevant DNA damage [3]. It was reported that the alternative sigma factor RpoS is an important element in the survival of *P. syringae* pv. *syringae* under natural sunlight [10]. Genes regulated by this protein have already been characterized in *E. coli* as involved in the cellular response to oxidative stress, including DNA repair and ROS quenching functions. Inactivation of *rpoS* lead to increased sensibility of *P. syringae* to solar UV, evidencing its role in UV tolerance for this organism [10].

REFERENCES

1 Coohill, T. P. & Sagripanti, J. L. Overview of the inactivation by 254 nm ultraviolet radiation of bacteria with particular relevance to biodefense. *Photochem Photobiol* **84**, 1084-1090, 10.1111/j.1751-1097.2008.00387.x (2008).

2 Slade, D. & Radman, M. Oxidative Stress Resistance in *Deinococcus radiodurans*. *Microbiol Mol Biol R* **75**, 133-191, 10.1128/Mmbr.00015-10 (2011).

3 Meador, J. A. *et al.* The significance of the Dewar valence photoisomer as a UV radiation-induced DNA photoproduct in marine microbial communities. *Environ Microbiol* **16**, 1808-1820, 10.1111/1462-2920.12414 (2014).

4 Feil, H. *et al.* Comparison of the complete genome sequences of *Pseudomonas syringae* pv. *syringae* B728a and pv. *tomato* DC3000. *P Natl Acad Sci USA* **102**, 11064-11069, 10.1073/pnas.0504930102 (2005).

5 Krisko, A. & Radman, M. Protein damage and death by radiation in *Escherichia coli* and *Deinococcus radiodurans*. *P Natl Acad Sci USA* **107**, 14373-14377, 10.1073/pnas.1009312107 (2010).

6 Santos, A. L. *et al.* Wavelength dependence of biological damage induced by UV radiation on bacteria. *Arch Microbiol* **195**, 63-74, 10.1007/s00203-012-0847-5 (2013).

7 Kim, J. J. & Sundin, G. W. Regulation of the *rulAB* mutagenic DNA repair operon of *Pseudomonas syringae* by UV-B (290 to 320 nanometers) radiation and analysis of *rulAB*-mediated mutability in vitro and in planta. *J Bacteriol* **182**, 6137-6144, Doi 10.1128/Jb.182.21.6137-6144.2000 (2000).

8 Sundin, G. W. & Murillo, J. Functional analysis of the *Pseudomonas syringae rulAB* determinant in tolerance to ultraviolet B (290–320 nm) radiation and distribution of *rulAB* among *P. syringae* pathovars. *Environ Microbiol* **1**, 75-87, 10.1046/j.1462-2920.1999.00008.x (1999).

9 Cazorla, F. M. *et al.* 62-kb plasmids harboring *rulAB* homologues confer UV-tolerance and epiphytic fitness to *Pseudomonas syringae* pv. *syringae* mango isolates. *Microb Ecol* **56**, 283-291, 10.1007/s00248-007-9346-7 (2008).

10 Miller, C. D., Mortensen, W. S., Braga, G. U. L. & Anderson, A. J. The *rpoS* gene in *Pseudomonas syringae* is important in surviving exposure to the near-UV in sunlight. *Curr Microbiol* **43**, 374-377, 10.1007/s002840010319 (2001).
